# Perceptions and practices of epinephrine use with local anesthetics in end-arterial areas among Jordanian physicians: A cross-sectional survey

**DOI:** 10.1097/MD.0000000000045068

**Published:** 2025-10-24

**Authors:** Marzouq Amarin, Ayah Elayan, Farah Bani-Khaled, Lana Shdaifat, Mahmoud Odeh, Omar Ismail, Daoud O. Al Aruri, Raed Al-Taher

**Affiliations:** a Department of General Surgery, School of Medicine, The University of Jordan, Amman, Jordan; b Department of General Surgery, Jordan University Hospital, The University of Jordan, Amman, Jordan; c School of Medicine, The University of Jordan, Amman, Jordan.

**Keywords:** end anatomical arteries, epinephrine, local anesthesia, physicians, WALANT

## Abstract

Epinephrine is frequently used in combination with local anesthetic agents to enhance their efficacy and duration of action. However, its use in anatomical regions supplied by end arteries remains a topic of controversy. The primary aim of this study was to evaluate physicians’ knowledge and practices regarding the use of epinephrine in combination with local anesthetics in end-arterial regions. We also aimed to estimate the prevalence of its use among Jordanian physicians and to examine variations in practice according to physician seniority and medical specialty. A cross-sectional study was conducted using a web-based questionnaire targeting Jordanian physicians. The questionnaire consisted of 3 sections that evaluated participants’ demographics, knowledge regarding epinephrine use with local anesthetics in end anatomical arteries, and practices related to general and local anesthesia when performing procedures on these areas. Data were analyzed using Jamovi 2.4.14 software. Of the 336 physicians, 56.5% were males, and 74.4% were aged 35 or younger. General surgery was the most common specialty (61.5%), with 74.7% of participants having <5 years of experience. Residents formed the majority (68.1%), while specialists comprised 31.9%. The overall knowledge about the safety of epinephrine with local anesthesia was low with scores of 3.53 ± 2.20, 2.11 ± 1.95, and 1.57 ± 2.20 for fingers/toes, nose/ears, and penile subgroups, respectively. Our results showed no statistical difference between junior and senior physicians in their choice between general and local anesthesia when operating on the penis (*P*-values = .564 and .571 for general anesthesia and local anesthesia, respectively). Also, hesitancy in using epinephrine was evident among fingers/toes and penile surgeons, being more obvious in the latter. Despite growing evidence supporting the safety of epinephrine in end-arterial regions, hesitancy remains obvious among Jordanian physicians, particularly in penile surgeries. Limited knowledge and variable practices across specialties highlight the need for targeted education and updated guidelines to fill the gap between evidence-based recommendations and clinical practices.

## 1. Introduction

The wide-awake local anesthesia no tourniquet (WALANT) technique, first implemented by Donald Lalonde in the early 2000’s, is a method that relies on local anesthetics and hemostatic agents to provide local anesthesia without sedation or using a tourniquet. This technique, widely adopted by hand surgeons, offers several advantages, including reduced intraoperative bleeding, minimal injection pain, absence of tourniquet-related discomfort, prolonged anesthetic duration, and decreased postoperative pain compared to regional or general anesthesia.^[1]^

Lidocaine, a widely used local anesthetic, has a rapid onset (<2 minutes), and a duration of action 1 to 2 hours. Its maximum dose is nearly 5 mg/kg.^[2]^ However, when combined with epinephrine, it slows the absorption of lidocaine into peripheral blood vessels, which prolongs the anesthetic effect into approximately 2 to 6 hours, with the most frequently reported duration being around 3 hours.^[3]^ Additionally, this combination increases the maximum allowable dose to 7 mg/kg.^[4]^

Commercially available formulations most commonly combine 1% or 2% lidocaine with epinephrine at concentrations of 1:100,000 (10 µg/mL) or 1:200,000 (5 µg/mL). Less frequently, stronger solutions such as 1:50,000 are used in dental and ENT surgeries. Epinephrine is usually not an optional adjunct in WALANT but serves as a fundamental aspect of the technique. Its vasoconstrictive effect minimizes bleeding, prolongs the duration of anesthesia, and eliminates the need for a tourniquet, which are features central to the WALANT technique. Despite these advantages, the use of local anesthetics mixed with epinephrine in areas of end-arteries such as the fingers, toes, penis, outer ear, and tip of the nose has been a controversial topic for decades. During the past century, it was common teaching that epinephrine should not be used in these areas due to fears of necrosis.^[5]^ Nonetheless, many studies have challenged this notion, with De Rougemont and Carcassoone demonstrating that digital necrosis can happen in hand surgeries even without using epinephrine,^[6]^ followed by providing 1500 cases of digital blocks with epinephrine, without digital necrosis. Several studies have then confirmed the safety of using local anesthesia with epinephrine in hand surgeries, with no systemic effects and no complications relating to ischemia and necrosis in the wounds being observed.^[7,8]^

The WALANT technique has also gained support in penile surgeries, offering benefits such as high patient satisfaction, relatively painless infiltration, and low complication rates.^[9]^ Additionally, studies on epinephrine use in ear and nose surgeries have demonstrated reduced blood perfusion without causing complete vascular blockade, thus preventing organ, tissue, or flap necrosis.^[10]^ Recently, WALANT technique has even been recognized as a simple, safe, and reliable method even in nipple reconstruction surgeries and skin flaps.^[11,12]^ Furthermore, in a comprehensive review of the literature of cases of necrosis, epinephrine itself was not believed to be the cause, with other possible contributing factors playing a role in causing necrosis. These include using older compounds (cocaine, eucaine, procaine), infection, tight tourniquet, and non-standardized inaccurate methods of mixing epinephrine with lidocaine.^[13]^ Similarly, a review of reported cases of digital necrosis and ischemia associated with epinephrine use found no consistent evidence linking commercially available lidocaine with epinephrine as the cause, instead supporting the theory of other contributing factors.^[14]^

Despite the evidence of the safety of using local anesthesia with epinephrine in end-arterial regions, hesitancy is still being observed among physicians, with a notable lack in literature exploring potential causes. In this study, our main objective was to investigate the understanding and current clinical practices among Jordanian physicians regarding the use of epinephrine with local anesthesia in regions supplied by terminal arteries.

## 2. Materials and methods

### 2.1. Study design and participants

We conducted a web-based cross-sectional study that aimed to address the following objectives^[1]^: to estimate the prevalence of local anesthetic with epinephrine use in end-arterial areas among Jordanian physicians;^[2]^ to evaluate their knowledge and attitudes toward this practice; and^[3]^ to examine differences according to physician seniority and specialty. A total of 336 physicians and surgeons participated in the study. Eligible participants included physicians in the following specialties: General surgery, urology, anesthesia, ENT, pediatric surgery, plastic surgery, and orthopedic surgery,^[2]^ residents, specialists, and consultants,^[3]^ Jordanian physicians practicing in Jordan or internationally,^[4]^ and participants who provided informed consent.

Participants were excluded if they met any of the following criteria: physicians from other specialties who did not perform procedures involving local anesthesia with epinephrine, and retired physicians.

This study was conducted and reported in accordance with the strengthening the reporting of observational studies in epidemiology guidelines.

### 2.2. Sample size

We adopted the convenience sampling method in our study. The Raosoft sample size calculator was used to estimate the sample size.^[15]^ With a 5% margin of error, a 95% confidence level, and a 50% response distribution, the sample size was calculated to be at least 335 participants.

A 50% response distribution was chosen because it represents the most conservative estimate, maximizing the required sample size. This assumption was made due to the limited available information regarding the expected response patterns in our target population.

Approximately 1000 physicians were approached to participate in the study in order to reach the target sample size. Of these, 336 completed the questionnaire, giving an estimated response rate of 33.6%.

### 2.3. Questionnaire development and validation

A questionnaire was developed by the research team based on existing literature regarding the safety and use of lidocaine with epinephrine. The questionnaire consisted of 3 main sections: sociodemographic information, knowledge regarding local anesthesia among physicians, and clinical practices. The sociodemographic section included age, gender, country of residence, professional level, specialty, and years of experience. The second section explored physicians’ knowledge regarding local analgesia and epinephrine use, which consisted of 8 questions for nose/ears surgeons group, and 9 questions for fingers/toes and penile surgeons groups. The third section assessed clinical practices among physicians taking into consideration years of experience. The questions of the third section were designed to allow the participant to choose one of the following: never, rarely, sometimes, very often, and always.

Participants were required to fill the first section. After that, they were given the choice to choose one or more areas of expertise including fingers and toes, nose and ears, and penis groups, where they will be required to answer the questions regarding knowledge and practices assigned to each subgroup.

To assess participants’ knowledge regarding the safety of epinephrine use in end-arterial regions, a scoring system was applied to the knowledge-related questions, with each question having one correct response. Responses were scored as the following: +1 for a correct answer, 0 for “I don’t know,” and –1 for an incorrect answer. Scores were calculated separately for each anatomical region. Positive scores reflected a favorable knowledge, whereas negative scores indicated an unfavorable knowledge.

Although the questionnaire was tailored using up-to-date clinical sources, it had not been previously validated. To ensure clarity, a pilot study was conducted, with face-to-face interviews, among 25 physicians. Feedback from participants was provided on readability, and ease of completion. No modifications were done following the pilot test. Cronbach alpha was calculated to assess the internal consistency of the knowledge items within each anatomical group. The fingers and toes group had a reliability coefficient of 0.67, indicating acceptable consistency. The nose and ears group demonstrated good internal consistency (α= 0.83), while the penile group showed excellent reliability (α = 0.92).

The questionnaire used in this study is provided as Supplementary File (Supplemental Digital Content, https://links.lww.com/MD/Q359)^[1]^.

### 2.4. Data collection

The questionnaire was created using google forms and was distributed through digital platforms, including e-mail and WhatsApp, to maximize reach among physicians from different specialties. Data collection began on March 1, 2024, and continued until December 20, 2024. Participation was entirely voluntary and anonymous, with no personal identifiers collected to ensure confidentiality. To prevent duplicate responses, participants were required to confirm that they had not previously completed the questionnaire before proceeding.

### 2.5. Data analysis

Data were analyzed using Jamovi 2.4.14 software. Descriptive statistics were used to summarize categorical variables, with results presented as frequencies, percentages, means, and standard deviations. Subgroup analyses were performed based on participants’ surgical specialties and years of experience to identify variations in practice patterns. *P*-value of <.05 was considered statistically significant for all analyses.

## 3. Results

### 3.1. Demographics

The questionnaire was filled by 336 physicians, 313 of them reported that they reside in Jordan (93.2%), and 190 were males (56.5%). About three-quarters were 35 years or younger (250, 74.4%), with a larger proportion being residents (68.1%) compared to specialists (31.9%). General surgery (61.6%) was the most prevalent specialty among the participants. Regarding experience, nearly three-quarters (74.7%) had 5 years or less of practice (Table 1).

**Table 1:** Demographic and professional characteristics of the participants.

Variable	n (%)
Gender	
Male	190 (56.5)
Female	146 (43.5)
Age	
25–35 yr	250 (74.4)
36–45 yr	45 (13.4)
>45 yr	41 (12.2)
Country of residence	
Inside Jordan	313 (93.2)
Outside Jordan	23 (6.8)
Professional level	
Resident	229 (68.1)
Specialist	107 (31.9)
Specialty	
Anesthesia	20 (6.0)
ENT	15 (4.5)
General surgery	207 (61.5)
Orthopedics	21 (6.3)
Pediatric surgery	23 (6.8)
Plastic surgery	21 (6.3)
Urology	29 (8.6)
Years of experience	
5 yr or less	251 (74.7)
More than 5 yr	85 (25.3)

### 3.2. Knowledge of epinephrine use in end-arterial regions

The knowledge of the participants who operate on the fingers and toes, nose and ears, and penis can be seen in Tables 2–4, respectively. While the majority of surgeons working on fingers and toes (88%) agreed that these are end-arterial areas, this view was less prevalent among ENT surgeons (68%) and penile surgeons (65%). More physicians acknowledged the advantages of using epinephrine in fingers and toes, with 76% stating that it reduces blood loss, compared to 61% for nose and ears and 43% for penile subgroups.

**Table 2:** Knowledge of the participants who operate on fingers and toes (N = 262 participant).

Statement	I don’t know	Disagree	Agree
Fingers and toes are considered areas of end artery	18 (7%)	13 (5%)	231 (88%)
Local anesthesia can be used instead of general anesthesia in these areas	46 (18%)	15 (5%)	201 (77%)
The addition of epinephrine to local anesthetics results in less blood loss from the site	49 (19%)	14 (5%)	199 (76%)
Epinephrine is safe to be mixed with local anesthetic agents in these sites	81 (31%)	73 (28%)	108 (41%)
Local anesthetic agents mixed with epinephrine decreases the need for using tourniquet in these sites	88 (34%)	25 (9%)	149 (57%)
Phentolamine can reverse the vasoconstrictive effect of epinephrine in fingers and toes	149 (57%)	9 (3%)	104 (40%)
The concentration of epinephrine in the anesthetic solution affects the risk of developing fingers and toes necrosis	77 (29%)	16 (6%)	169 (65%)
The total volume of epinephrine in the anesthetic solution affects the risk of developing fingers and toes necrosis	91 (35%)	23 (9%)	148 (56%)
Epinephrine mixed with local anesthetics is safe to use in patients with preexisting conditions like sever scleroderma, Buerger disease, or generalized atherosclerosis.	99 (38%)	136 (52%)	27 (10%)
Score (mean ± SD)	3.53 ± 2.2		

Reported as frequencies (percentages), positive score reflects positive knowledge and negative score reflects negative knowledge.

**Table 3:** Knowledge of the participants who operate on nose and ears (N = 272 participant).

Statement	I don’t know	Disagree	Agree
Nose and ears are considered areas of end artery	53 (19%)	33 (13%)	186 (68%)
Local anesthesia can be used instead of general anesthesia in these areas	95 (35%)	41 (15%)	136 (50%)
The addition of epinephrine to local anesthetics results in less blood loss from the site	93 (34%)	12 (5%)	167 (61%)
Epinephrine is safe to be mixed with local anesthetic agents in these sites	122 (45%)	43 (16%)	107 (39%)
Phentolamine can reverse the vasoconstrictive effect of epinephrine in nose and ears	175 (64%)	6 (2%)	91 (34%)
The concentration of epinephrine in the anesthetic solution affects the risk of developing nose and ears necrosis	117 (43%)	11 (4%)	144 (53%)
The total volume of Epinephrine in the anesthetic solution affects the risk of developing nose and ears necrosis	130 (48%)	23 (9%)	119 (43%)
Epinephrine mixed with local anesthetics is safe to use in patients with preexisting conditions like sever scleroderma, Buerger disease, or generalized atherosclerosis.	139 (51%)	96 (35%)	37 (14%)
Score (mean ± SD)	2.11 ± 1.95		

Reported as frequencies (percentages), positive score reflects positive knowledge and negative score reflects negative knowledge.

**Table 4:** Knowledge of the participants who operate on penis (N = 323 participant).

Statement	I don’t know	Disagree	Agree
The penis is considered an area of end artery	100 (31%)	13 (4%)	210 (65%)
local anesthesia can be used instead of general anesthesia in this area	156 (48%)	60 (19%)	107 (33%)
The addition of epinephrine to local anesthetics results in less blood loss from the site	152 (47%)	32 (10%)	139 (43%)
Epinephrine is safe to be mixed with local anesthetic agents in this site	175 (58%)	67 (22%)	58 (20%)
Local anesthetic agents mixed with epinephrine decreases the need for using tourniquet in this site	192 (60%)	38 (11%)	93 (29%)
Phentolamine can reverse the vasoconstrictive effect of epinephrine in the penis	209 (65%)	17 (5%)	97 (30%)
The concentration of epinephrine in the anesthetic solution affects the risk of developing penile necrosis	163 (50%)	14 (5%)	146 (45%)
The total volume of Epinephrine in the anesthetic solution affects the risk of developing penile necrosis	169 (52%)	31 (10%)	123 (38%)
Epinephrine mixed with local anesthetics is safe to use in patients with preexisting conditions like sever scleroderma, Buerger disease, or generalized atherosclerosis.	178 (55%)	108 (34%)	37 (11%)
Score (mean ± SD)	1.57 ± 2.2		

Reported as frequencies (percentages), positive score reflects positive knowledge and negative score reflects negative knowledge.

Confidence regarding the safety of using epinephrine in these areas was highest for fingers and toes (41%), followed by nose and ears (39%), and penile procedures (20%). When inquiring whether adding epinephrine reduces the need for a tourniquet, 57% of surgeons in the fingers and toes group agreed, while uncertainty remained the prevalent view in penile surgeons with 60% being unsure. In terms of reversing the vasoconstrictive effects of epinephrine with phentolamine, 40% of the finger and toes group agreed on its efficacy, compared to 34% of nose and ears surgeons, and 30% of penile surgeons group.

In the fingers and toes subgroup, 65% of participants believed that the concentration of epinephrine influences the risk of necrosis, while 56% thought that the volume of the solution contributes to this risk. Among nose and ears surgeons, 53% recognized the effect of concentration, while 43% acknowledged the impact of volume. In the penile group, more participants agreed on the effect of concentration (45%) compared to the volume of the solution (38%) in relation to necrosis risk. However, uncertainty regarding the role of concentration and volume remained the dominant belief. When discussing the safety of using epinephrine in patients with preexisting conditions like scleroderma and Buerger’s disease, only 10%, 14%, and 11% agreed that mixing epinephrine with the local anesthetic is safe in patients with these conditions in fingers and toes, nose and ears, and penile groups, respectively.

The knowledge scores revealed overall low level of knowledge. Participants who operate on the fingers and toes had the highest average knowledge score of 3.53 ± 2.20. Surgeons operating on the nose and ears scored an average of 2.11 ± 1.95, while those performing penile surgeries had the lowest knowledge score of 1.57 ± 2.20.

### 3.3. Practices in epinephrine use for end-arterial surgeries

Physician practices regarding epinephrine use and anesthesia selection for fingers and toes, nose and ears, and penis are represented in Tables 5–7, respectively. Results varied by surgical site and experience level, as more experienced physicians (>5 years) were significantly more likely to prefer local anesthesia for fingers and toes (*P*-value < .001), and nose and ears (*P*-value < .001) surgeries. However, experience among surgeons who operate on the penis had no significant impact on anesthesia choice (*P*-values = .564 and .571 for general anesthesia and local anesthesia, respectively).

**Table 5:** Practices of the participants who operate on fingers and toes, and the association with their years of experience.

Experience	Never	Rarely	Sometimes	Very often	Always	*P*-value
How often do you use general anesthesia						
5 yr or less	107 (51%)	43 (20%)	41 (20%)	11 (5.2%)	8 (3.8%)	<.001*
More than 5 yr	5 (10%)	8 (15%)	21 (40%)	17 (33%)	1 (2%)	
How often do you use local anesthesia						
5 yr or less	102 (49%)	7 (3 %)	24 (11%)	57 (27%)	20 (10%)	<.001*
More than 5 yr	3 (6%)	2 (4%)	16 (31%)	25 (47%)	6 (12%)	
How often do you mix epinephrine with local anesthetic agents						
5 yr or less	138 (65%)	14 (7%)	35 (17%)	12 (6%)	11 (5%)	.230
More than 5 yr	29 (56%)	7 (13%)	7 (13%)	6 (12%)	3 (6%)	

Total counts are reported as frequencies (percentages).

* Significant at *P*-value <.05.

**Table 6:** Practices of the participants who operate on nose and ears, and the association with their years of experience (n = 272).

Experience	Never	Rarely	Sometimes	Very often	Always	*P*-value
How often do you use general anesthesia						
5 yr or less	125 (58%)	8 (4%)	24 (11%)	43 (20%)	16 (7%)	.005*
More than 5 yr	19 (34%)	2 (3%)	6 (11%)	24 (43%)	5 (9%)	
How often do you use local anesthesia						
5 yr or less	132 (61%)	15 (7%)	34 (16%)	24 (11%)	11 (5%)	<.001*
More than 5 yr	22 (39%)	1 (2%)	7 (13%)	17 (30%)	9 (16%)	
How often do you mix epinephrine with local anesthetic agents						
5 yr or less	149 (69%)	19 (9%)	17 (8%)	18 (8%)	13 (6%)	<.001*
More than 5 yr	27 (47%)	2 (4%)	2 (4%)	15 (27%)	10 (18%)	

Total counts are reported as frequencies (percentages).

* Significant at *P*-value <.05.

**Table 7:** Practices of the participants who operate on the penis and the association with their years of experience (N = 323).

Experience	Never	Rarely	Sometimes	Very often	Always	*P*-value
How often do you use general anesthesia						
5 yr or less	155 (63%)	2 (1%)	22 (9%)	38 (16%)	28 (11%)	.564
More than 5 yr	37 (47%)	0 (0%)	6 (8%)	16 (21%)	19 (24%)	
How often do you use local anesthesia						
5 yr or less	165 (67%)	27 (11%)	38 (16%)	7 (3%)	8 (3%)	.571
More than 5 yr	43 (55%)	14 (18%)	13 (17%)	7 (9%)	1 (1%)	
How often do you mix epinephrine with local anesthetic agents						
5 yr or less	198 (81%)	18 (7%)	16 (7%)	8 (3%)	5 (2%)	.561
More than 5 yr	65 (84%)	5 (6%)	4 (5%)	4 (5%)	0 (0%)	

Total counts are reported as frequencies (percentages).

When asked about the use of epinephrine with lidocaine in their practice, the majority of participants across the 3 groups reported never using it, with this view being more obvious across penile surgeons as more than 80% of physicians reported never using it. Experience had no effect on epinephrine use in fingers and toes surgeries (*P*-value = .230) and nose and ears surgeries (*P*-value = .561). However, the role of experience was evident among nose and ear surgeons where more experienced physicians were more likely to use epinephrine in their procedures (*P*-value < .001).

## 4. Discussion

This study aimed to evaluate current knowledge of physicians and surgeons regarding the use of epinephrine with lidocaine in surgical sites with end anatomical arteries, as well as to explore current practices of anesthesia choices. While earlier research has addressed the advantages and disadvantages of using epinephrine with local anesthesia, this is the first study to evaluate the present situation and knowledge among Jordanian doctors.

Although knowledge average scores showed a positive reflection, they were relatively low, with the highest average score noted among finger and toes group (3.53). Surgeons operating on the nose and ears scored an average of 2.11, while those performing penile surgeries had the lowest knowledge score of 1.57. This reflects a low knowledge level among physicians in Jordan, which may be related to practice patterns during residency, which means lower exposure to WALANT technique, and possible fear of complications related to epinephrine use.

When assessing levels of knowledge, the majority of participants across the 3 groups agreed that fingers, toes, penis, ears, and nose are areas of end anatomical arteries. However, variations about the safety of epinephrine in these sites were observed. Around 40% of fingers/toes and nose/ears surgeons agreed on the safety of using epinephrine. Although the percentage is relatively low, several studies had confirmed epinephrine’s safety, particularly in hand surgeries.^[16,17]^ In regard to nose and ears surgeries, Häfner et al documented the administration of epinephrine with local anesthesia in over 10,000 ear and nose surgeries, using concentrations ranging from 1:1000,000 to commercially available 1:200,000, without encountering complications in any patient.^[10]^ Moreover, a retrospective study conducted in Egypt also highlighted the safety of epinephrine use in ear surgeries across preparations containing adrenaline at 1:20,000 to 1:200,000 concentrations, which was described as an excellent hemostatic agent. This study also had no documented local ischemic complications.^[18]^

In contrast to fingers/toes and nose/ears surgeons, penile surgeons demonstrated uncertainty regarding the safety of epinephrine, as 58% stated that they do not know. Reviewing the literature reveals a controversy surrounding the safety of epinephrine usage in penile surgeries. One prospective study evaluated the safety and efficacy of using epinephrine (1:100,000) in hypospadias surgeries in Jordan. Apart from affirming its safety, the study highlighted several advantages including less need for a tourniquet, less utilization of bipolar diathermy, less operative time, and shorter hospital stay.^[19]^ However, isolated cases of penile and scrotal skin necrosis after circumcision have been reported. In one such case, 1% lidocaine with epinephrine 1:200,000 was injected subcutaneously using a ring block technique.^[20,21]^ Nevertheless, the literature has not yet established a direct causal relationship between epinephrine use and penile ischemia. Moreover, 57% of participants operating on fingers and toes agreed that using epinephrine decreases the need for tourniquets, thus reducing possible complications associated with it. These findings align with trials done using the WALANT technique in hand surgeries, resulting in reduced pain and bleeding, along with a decreased need for tourniquet use and the pain associated with it.^[3,22]^

When assessing levels of knowledge about the effects of epinephrine’s concentration and volume on the risk of necrosis, participants perceived the concentration to have a slightly greater impact than the total volume administered. Several studies have investigated the relationship between different epinephrine concentrations and their vasoconstrictor effects, including the risk of necrosis. Kermani et al found no significant difference in bleeding levels during rhinoplasty surgeries when comparing different concentrations of lidocaine and epinephrine (1:100,000 vs 1:200,000), suggesting no disruption in blood flow to the nose.^[23]^ Similarly, Gessler et al reported that there was no significant difference between 1:50,000, 1:100,000, and 1:200,000 epinephrine-containing solutions in reducing blood flow in ear surgeries.^[24]^ In regard to the potential risk of necrosis, Steinberg and Block reported their observations in 1971 in over 200,000 patients undergoing forefoot and toe surgeries. They reported no cases of necrosis or gangrene when using low concentration of epinephrine combined with lidocaine (1:100,000–1:200,000).^[17]^ A large multicentric study by Lalonde et al confirmed the safety of low-dose epinephrine (1:100,000 or less) among 3110 cases of elective hand surgeries with no observed cases of digital necrosis.^[16]^ Even though existing literature supports the safety of low doses of epinephrine in end anatomical arteries, there are still some reported cases of epinephrine-related necrosis, with most of them being attributed to factors other than the concentration.^[25]^

Unlike concentration, the volume of epinephrine has been shown to increase the risk of potential necrosis in end anatomical arteries. In 2001, Denkler conducted a review of the literature on digital necrosis following local anesthesia and found only 48 cases reported over 120 years, with epinephrine involved in 21 cases. Importantly, some historical preparations reached concentrations as high as 1:1000, much higher than those used in contemporary practice. Various factors were identified, but one of them was the larger volume of epinephrine than usual.^[14]^ In 2017, Zhu et al also reported a case of finger ischemia following the administration of local anesthesia with epinephrine, which was attributed to the volume of the mixture.

Furthermore, when discussing the safety of using epinephrine in patients with preexisting conditions like scleroderma, Buerger’s disease, and generalized atherosclerosis, nearly half of the surgeons in both the ENT and penile groups indicated that they did not know. Conversely, 52% of surgeons in the finger/toes group disagreed with the use of epinephrine in such cases, likely reflecting greater exposure to vascular conditions, which often present with manifestations in both the upper and lower extremities. These concerns align with the literature, as there are reported cases of digital necrosis following epinephrine use in patients with preexisting conditions. Two of these cases describe digital gangrene following the administration of epinephrine 1:100,000 in patients with Raynaud’s phenomenon.^[26,27]^ Additionally, Hutting et al reported a case of digital necrosis in a diabetic patient after using epinephrine with a concentration of 1:100,000. Further investigations revealed severe atherosclerotic changes in the affected hand.^[28]^ Thus, it is generally advised to avoid epinephrine use in patients with vascular diseases.

Our findings also revealed significant variability in anesthesia and epinephrine use among physicians. More experienced physicians were more likely to use local anesthesia over general anesthesia in fingers and toes and nose and ear surgeries. This aligns with the increasing trend toward WALANT techniques, especially following Coronavirus Disease 2019 pandemic, during which, the least physician-patient contact was required to minimize viral spread.^[29,30]^ However, less experienced physicians preferred general anesthesia, likely due to lack of exposure to WALANT technique during training, or concerns about potential complications. Interestingly, when it came to penile surgeries, years of experience did not significantly influence anesthesia choices. Despite emerging literature supporting the use of epinephrine in penile procedures,^[9,19]^ fears about vascular compromise and necrosis may contribute to its limited use.

When examining epinephrine use, our results showed that experience significantly influenced its involvement in nose and ear surgeries, where more experienced physicians were more confident using epinephrine, indicating a higher level of adherence to emerging evidence.^[31]^ However, in fingers, toes, and penile procedures, experience did not play a significant role. This was more obvious among penile surgeons where more than 80% of junior and senior physicians admitted not using epinephrine in their procedures. This suggests that hesitation toward epinephrine use in these regions, particularly the penis, persists across all levels of expertise, likely due to long-standing concerns about ischemia and necrosis. Several studies have highlighted the benefits of using epinephrine in ear/nose and finger/toe surgeries, however, the reluctance to use epinephrine in penile surgeries may reflect a lack of similar evidence supporting its safety, influencing physicians to avoid it more often.

According to the existing literature, guidance can be drawn regarding the safe use of lidocaine with epinephrine in end-arterial areas. For digital blocks, large prospective studies confirm the safety of lidocaine with epinephrine at 1:100,000 or 1:200,000 concentrations, with no reports of necrosis using commercially prepared solutions. In the ear and nasal regions, both retrospective and prospective cohorts document safe use across concentrations ranging from 1:100,000 to 1:200,000, and some studies have even reported the safe use of more concentrated preparations such as 1:50,000 and 1:20,000. For penile blocks, published data most often describe lidocaine with epinephrine 1:100,000 or less, with overall safety but occasional case reports of necrosis highlighting the importance of careful technique and limited volume. By contrast, the historical use of highly concentrated solutions (e.g., 1:1000) has been linked to adverse outcomes and should not be used in current practice. Taken together, the evidence supports the use of commercially available formulations in the 1:100,000 to 1:200,000 range, within a maximum lidocaine dose of ≤7 mg/kg, while maintaining caution in patients with significant vascular compromise. This framework provides clinicians with safe, evidence-based parameters for everyday practice.

### 4.1. Strengths and limitations

Our study is the first study in Jordan and the region to assess physicians’ knowledge and practices regarding the use of epinephrine in end-arterial regions. Additionally, this study utilized a cross-sectional survey design, allowing for a broad assessment of physicians’ knowledge and practices regarding epinephrine use in end-artery regions. The questionnaire was structured based on existing clinical guidelines, ensuring relevance to current medical practice. The study also included a large and diverse sample of physicians from multiple specialties, enhancing the generalizability of findings. However, our study design poses some limitations. Self-reported data may be subject to recall bias, and reporting the practices may be influenced by social desirability bias potentially affecting the accuracy of reported knowledge and practices. The estimated response rate was 33.6%, which is considered acceptable for web-based physician surveys. However, the possibility of non-response bias cannot be excluded and may affect the generalizability of the findings. Finally, the study used a non-probability sampling method, which may limit the generalizability of the findings.

## 5. Conclusion

In conclusion, our results demonstrate low level of knowledge and acceptance of local anesthesia despite growing evidence supporting the safe use of epinephrine in end-arterial regions. Longstanding beliefs continue to influence clinical decision-making. Our findings suggest that education, more than experience alone, is critical in spreading knowledge about the safety of epinephrine use in end-anatomical arteries. Bridging this gap through targeted training, particularly during residency programs, and updated guidelines is essential to align practice with up-to-date evidence.

## Acknowledgments

The authors appreciate the time spent by all the respondents for filling the questionnaire and thank them for their participation.

## Author contributions

**Conceptualization:** Marzouq Amarin.

**Data curation:** Ayah Elayan, Farah Bani-Khaled, Lana Shdaifat, Mahmoud Odeh.

**Formal analysis:** Omar Ismail, Daoud O. Al Aruri.

**Methodology:** Marzouq Amarin.

**Supervision:** Raed Al-Taher.

**Validation:** Omar Ismail, Daoud O. Al Aruri.

**Writing – original draft:** Ayah Elayan, Farah Bani-Khaled, Lana Shdaifat, Mahmoud Odeh.

**Writing – review & editing:** Marzouq Amarin, Omar Ismail, Daoud O. Al Aruri, Raed Al-Taher.

## Supplementary Material


