# Supplementary material for: Perceptions and practices of epinephrine use with local anesthetics in end-arterial areas among Jordanian physicians: A cross-sectional survey
Source: Medicine (Baltimore). 2025 Oct 24;104(43):e45068. doi: 10.1097/MD.0000000000045068 (PMC12558206; doi:10.1097/MD.0000000000045068)
Supplement: Supplementary file 1 [file medi-104-e45068-s001.docx]

# Questionnaire: Knowledge and Practices Regarding Local Anesthetic with Epinephrine in End-Arterial Areas

## Section 1: Demographics & General Practice

1. Gender*
- Male
- Female

2. Age group*
- 25–35
- 36–45
- 46–55
- 56–65
- ≥65
- Other: ____________

3. Current country of residence*
[Dropdown of countries]

4. Professional level*
- Resident
- Specialist
- General practitioner

5. Specialty*
- General Surgery
- Urology
- Anesthesia
- ENT
- Pediatric Surgery
- Plastic Surgery
- General Practice
- Orthopaedic Surgery

6. Years of clinical experience*
[Number]

## Section 2: Knowledge

Please indicate your level of agreement with the following statements (Answer options: I don't know, Disagree, Agree).

### Fingers & Toes

Fingers and toes are considered areas of end artery.
- I don't know
- Disagree
- Agree

Local anesthesia can be used instead of general anesthesia in these areas.
- I don't know
- Disagree
- Agree

The addition of epinephrine to local anesthetics results in less blood loss from the site.
- I don't know
- Disagree
- Agree

Epinephrine is safe to be mixed with local anesthetic agents in these sites.
- I don't know
- Disagree
- Agree

Local anesthetic agents mixed with epinephrine decreases the need for using tourniquet in these sites.
- I don't know
- Disagree
- Agree

Phentolamine can reverse the vasoconstrictive effect of epinephrine in fingers and toes.
- I don't know
- Disagree
- Agree

The concentration of epinephrine in the anesthetic solution affects the risk of developing fingers and toes necrosis.
- I don't know
- Disagree
- Agree

The total volume of epinephrine in the anesthetic solution affects the risk of developing fingers and toes necrosis.
- I don't know
- Disagree
- Agree

Epinephrine mixed with local anesthetics is safe to use in patients with pre-existing conditions like severe scleroderma, Buerger disease, or generalized atherosclerosis.
- I don't know
- Disagree
- Agree

### Nose & Ears

Nose and ears are considered areas of end artery.
- I don't know
- Disagree
- Agree

Local anesthesia can be used instead of general anesthesia in these areas.
- I don't know
- Disagree
- Agree

The addition of epinephrine to local anesthetics results in less blood loss from the site.
- I don't know
- Disagree
- Agree

Epinephrine is safe to be mixed with local anesthetic agents in these sites.
- I don't know
- Disagree
- Agree

Phentolamine can reverse the vasoconstrictive effect of epinephrine in nose and ears.
- I don't know
- Disagree
- Agree

The concentration of epinephrine in the anesthetic solution affects the risk of developing nose and ears necrosis.
- I don't know
- Disagree
- Agree

The total volume of epinephrine in the anesthetic solution affects the risk of developing nose and ears necrosis.
- I don't know
- Disagree
- Agree

Epinephrine mixed with local anesthetics is safe to use in patients with pre-existing conditions like severe scleroderma, Buerger disease, or generalized atherosclerosis.
- I don't know
- Disagree
- Agree

### Penile Procedures

The penis is considered an area of end artery.
- I don't know
- Disagree
- Agree

Local anesthesia can be used instead of general anesthesia in this area.
- I don't know
- Disagree
- Agree

The addition of epinephrine to local anesthetics results in less blood loss from the site.
- I don't know
- Disagree
- Agree

Epinephrine is safe to be mixed with local anesthetic agents in this site.
- I don't know
- Disagree
- Agree

Local anesthetic agents mixed with epinephrine decreases the need for using tourniquet in this site.
- I don't know
- Disagree
- Agree

Phentolamine can reverse the vasoconstrictive effect of epinephrine in the penis.
- I don't know
- Disagree
- Agree

The concentration of epinephrine in the anesthetic solution affects the risk of developing penile necrosis.
- I don't know
- Disagree
- Agree

The total volume of epinephrine in the anesthetic solution affects the risk of developing penile necrosis.
- I don't know
- Disagree
- Agree

Epinephrine mixed with local anesthetics is safe to use in patients with pre-existing conditions like severe scleroderma, Buerger disease, or generalized atherosclerosis.
- I don't know
- Disagree
- Agree

## Section 4: Practices

Please indicate how often you perform the following practices (Answer options: Never, Rarely, Sometimes, Very often, Always).

### Fingers & Toes

How often do you use general anesthesia in surgeries involving fingers/toes?
- Never
- Rarely
- Sometimes
- Very often
- Always

How often do you use local anesthesia in surgeries involving fingers/toes?
- Never
- Rarely
- Sometimes
- Very often
- Always

How often do you mix epinephrine with local anesthetic agents in these surgeries?
- Never
- Rarely
- Sometimes
- Very often
- Always

### Nose & Ears

How often do you use general anesthesia in surgeries involving nose/ears?
- Never
- Rarely
- Sometimes
- Very often
- Always

How often do you use local anesthesia in surgeries involving nose/ears?
- Never
- Rarely
- Sometimes
- Very often
- Always

How often do you mix epinephrine with local anesthetic agents in these surgeries?
- Never
- Rarely
- Sometimes
- Very often
- Always

### Penile Procedures

How often do you use general anesthesia in penile surgeries?
- Never
- Rarely
- Sometimes
- Very often
- Always

How often do you use local anesthesia in penile surgeries?
- Never
- Rarely
- Sometimes
- Very often
- Always

How often do you mix epinephrine with local anesthetic agents in these surgeries?
- Never
- Rarely
- Sometimes
- Very often
- Always
